# Optimized high‐fidelity 3DPCR to assess potential mitochondrial targeting by activation‐induced cytidine deaminase

**DOI:** 10.1002/2211-5463.12927

**Published:** 2020-08-13

**Authors:** Haiyan Wu, Kaili Zhang, Yue Chen, Jinfeng Li, Matthew P. Strout, Xiwen Gu

**Affiliations:** ^1^ Key Laboratory of Shaanxi Province for Craniofacial Precision Medicine Research Research Center of Stomatology Xi'an Jiaotong University College of Stomatology Xi'an China; ^2^ Department of Oral and Maxillofacial Surgery Xi'an Jiaotong University College of Stomatology Xi'an China; ^3^ Department of Periodontology and Oral Medicine Xi'an Jiaotong University College of Stomatology Xi'an China; ^4^ Section of Hematology Yale University School of Medicine New Haven CT USA; ^5^ Present address: Alexion Pharmaceuticals New Haven CT USA

**Keywords:** 3DPCR, activation‐induced cytidine deaminase, mitochondria, mtDNA

## Abstract

Activation‐induced cytidine deaminase (AID) initiates somatic hypermutation and class switch recombination of immunoglobulin genes in B cells, whereas off‐targeted AID activity contributes to oncogenic mutations and chromosomal translocations associated with B cell malignancies. Paradoxically, only a minority of AID is allowed to access the nuclear genome, but the majority of AID is retained in the cytoplasm. It is unknown whether cytoplasmic AID can access and target the mitochondrial genome [mitochondrial DNA (mtDNA)]. To address this issue, we developed high‐fidelity differential DNA denaturation PCR, which allowed the enrichment of genuine mtDNA mutations and therefore the identification of endogenous mtDNA mutation signatures *in vitro*. With this approach, we showed that AID targeting to mtDNA is a rare event in AID‐expressing lymphoma lines. Further biochemical and microscopic analysis revealed that a fraction of cytosol AID is associated with the outer membrane of mitochondria but unable to access the mitochondrial matrix. Together, our data suggested that the mitochondrial genome is protected from AID‐mediated mutagenesis by physical segregation of AID from accessing mtDNA within the mitochondrial matrix.

## Graphical abstract

Here, we develop a high‐fidelity differential DNA denaturation PCR approach and investigate whether activation‐induced cytidine deaminase (AID) can target mitochondrial DNA (mtDNA) *in vitro*. We demonstrate AID targeting of mtDNA is a rare event. Despite a fraction of AID binding the mitochondrial outer membrane, it is unable to access the mitochondrial matrix or the mtDNA. Thus, the mitochondrial genome is protected from AID‐mediated mutagenesis by physical segregation.

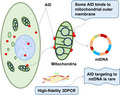

**Abbreviations**
3DPCR: differential DNA denaturation PCR
AID: activation‐induced cytidine deaminase
Ig: immunoglobulin
mtDNA: mitochondrial DNA

Activation‐induced cytidine deaminase (AID) is a single‐strand DNA deaminase with versatile and crucial functions. In B lymphocytes, AID initiates somatic hypermutation and class switch recombination of immunoglobulin (Ig) genes via deamination of cytosine (C) into uracil (U). The resulting U:G (guanine) mismatches are subsequently processed by downstream base excision and DNA mismatch repair pathways to yield DNA mutations and strand lesions [1, 2]. Promiscuous off‐targeting AID activity has been shown to cause oncogenic point mutations, DNA double‐strand breaks and chromosome translocations associated with B cell malignancies [3, 4, 5, 6]. In addition, inflammatory response can trigger ectopic expression of AID in non‐B cells, and AID‐associated mutagenesis has been implicated in the pathogenesis of pancreatic, gastric, colon and skin cancers [7, 8, 9, 10]. Furthermore, several studies have found that AID can function as a critical epigenetic regulator in a great variety of cell types, including B cells, hematopoietic stem cells and embryonic stem cells [11, 12, 13, 14, 15].

Restraining AID access to the nucleus has emerged as one major mechanism to curb deleterious AID activity. For instance, nuclear AID has been shown to be susceptible to proteasomes reactivator REG‐gamma‐mediated degradation or is subjected to CRM1‐dependent active nuclear export [16, 17]. As a result, AID targeting of nuclear DNA is transient, and the majority of AID is localized within the cytoplasm [18, 19, 20]. It remains unknown whether cytoplasmic AID plays a role other than sequestering AID from the nuclear genome. In this regard, mitochondria are cytoplasmic organelles central for oxidative phosphorylation, fatty acid oxidation and apoptosis signaling. Despite major mitochondrial proteins being encoded by nuclear DNA, mitochondria contain their own mitochondrial genome [mitochondrial DNA (mtDNA)] within the mitochondrial matrix. The circular mtDNA in human consists of 16 569 bp and encodes 13 key components of the mitochondrial respiratory chain complex. It is unknown whether mtDNA is targeted by or protected from AID‐mediated mutagenesis.

Here, we developed high‐fidelity differential DNA denaturation PCR (3DPCR) to investigate mtDNA mutations in AID‐expressing lymphoma cell lines and revealed little evidence for AID targeting of mtDNA. Further biochemical analysis revealed that a fraction of AID is associated with the outer membrane of mitochondria but is protected from accessing the mitochondrial matrix and mtDNA within it, suggesting that mtDNA is protected from AID‐mediated mutagenesis by physical segregation.

## Materials and methods

### Cell culture

Human Burkitt lymphoma cell line Ramos [21] and Ramos‐A23 (a Ramos‐derived line with constitutive high expression of AID driven by overexpression construct) were cultured in RPMI‐1640 (BE04‐558F; Lonza, Allendale, NJ, USA) supplemented with 10% FBS (HyClone, Cramlington, UK), 2 mm l‐glutamine and 1% penicillin/streptomycin (10378016; Thermo, Waltham, MA, USA). Human chronic B cell leukemia line Mec1 was cultured in Iscove’s modified Dulbecco’s medium (12‐722F; Lonza) supplemented with 10% FBS, 2 mm l‐glutamine and 1% penicillin/streptomycin. Chicken DT40 and DT40AID^−/−^ cells [22] were cultured in RPMI‐1640 supplemented with 10% FBS, 1% chicken serum (C5405; Sigma, St. Louis, MO, USA), 2 mm l‐glutamine, 1% penicillin/streptomycin and 0.1 mm β‐mercaptoethanol. 293T cells were cultured in Dulbecco’s modified Eagle’s medium (12‐604F; Lonza) supplemented with 10% FBS, 2 mm l‐glutamine and 1% penicillin/streptomycin. All cultures were maintained at 37 °C in a humidified tissue culture incubator with 5% CO_2_.

### Retroviral transfection and GFPstop reporter assay

293T cells were cotransfected with the pKAT2 retroviral packaging plasmid and a viral vector encoding an inactive GFP (MMLV–GFPstop–IRES–Puro) [23] using Lipofectamine 3000 reagent (Invitrogen, Waltham, MA, USA) following the manufacturer’s protocol. Two days posttransfection, viral supernatant was harvested and added to Ramos, Ramos‐A23 and Mec1 cells, followed by centrifugation at room temperature (420 ***g*** for 45 min). Forty‐eight hours postinfection, retrovirus‐transduced cells were selected for 3 weeks with optimal concentration of puromycin (0.25 μg·mL^−1^ for Ramos and Ramos‐A23 cells and 1 μg·mL^−1^ for Mec1 cells). To detect GFP reversion mutants, we resuspended 1 × 10^6^ transduced or control cells in PBS and evaluated for GFP expression with a FACSCalibur flow cytometer using cellquest software (BD, Franklin Lakes, NJ, USA).

### Immunofluorescence analysis

Where indicated, cells were stained with 200 nm MitoTracker Red (Invitrogen) in RPMI‐1640 for 15 min at 37 °C. Cells were then fixed and permeabilized at −20 °C with prechilled 100% methanol for 10 min. After three washes with PBS, nonspecific antibody binding was blocked with 5% goat serum in PBS. The cells were then incubated for 1 h with mouse anti‐AID IgG1 (1 : 100; 392500; Invitrogen) primary antibody. After three washes with PBS, the cells were incubated with Alexa Fluor 488‐conjugated goat anti‐(mouse IgG1) (1 : 200; Invitrogen) secondary antibodies for 1 h. Nuclei were counterstained with DAPI‐containing mounting medium (Vector Shield). Immunofluorescence images were captured using a Nikon eclipse‐TI confocal microscopy (Perkin Elmer, Walthan, MA, USA).

### Mitochondria treatment and western blot analysis

Mitochondria were purified from 2 × 10^7^ cultured cells using Qproteome mitochondria isolation kit (Qiagen, Hilden, Germany) following the manufacturer’s protocol, which allows simultaneous isolation of cytosol and mitochondrial proteins. For proteinase digestion, isolated mitochondria were incubated with 5–50 µg·mL^−1^ trypsin on ice for 30 min. Samples were separated by SDS/PAGE, transferred to poly(vinylidene difluoride) membranes and subsequently probed with mouse anti‐AID IgG1 (1 : 1000; 392500; Invitrogen), rabbit anti‐VDAC (Voltage‐dependent anion channel) Ig (1 : 1000; 4866; Cell Signaling, Danvers, MA, USA), rabbit anti‐β‐Tubulin Ig (1 : 2000; sc‐9104; Santa Cruz) and rabbit anti‐Hsp60 Ig (1 : 1000; 4870; Cell Signaling) primary antibodies, followed by corresponding goat anti‐(rabbit Ig) (1 : 5000; SC‐2004; SCBT, Santa Cruz, CA, USA) and rabbit anti‐(mouse Ig) (1 : 10 000; AB_2340074; Jackson Immuno, West Grove, PA, USA) secondary antibodies coupled to horseradish peroxidase according to the manufacturer’s protocol.

### 3DPCR error rate

An 897‐bp Cytb product was amplified from Ramos cells, cloned into pMD19‐T vector (pMD19‐Cytb) and used as a template for evaluating 3DPCR error spectrum. For Taq‐based two‐step 3DPCR, the first‐round standard PCR was amplified using 1 U Taq polymerase (Takara, Dalian, China), 1 ng pMD19‐Cytb template and M13 universal primers (M13F, 5′‐TGTAAAACGACGGCCAGT‐3′; M13R, 5′‐CAGGAAACAGCTATGACC‐3′), according to the following program: 95 °C for 5 min, 30 cycles at 95 °C for 30 s, 53 °C for 30 s, 72 °C for 60 s and a final extension step of 72 °C for 10 min. The second‐round 3DPCR was amplified using 1 U Taq, 1/50 of the first‐round product and Cytb primers (CytbF, 5′‐AACCATCGTTGTATTTCAAC‐3′; CytbR, 5′‐TAGTTTGTTAGGGACGGATC‐3′), according to the following program: 95 °C for 5 min, 35 cycles at 85–90 °C for 30 s, 53 °C for 30 s, 72 °C for 60 s and a final extension step of 72 °C for 10 min. Taq‐based single 3DPCR was performed following the same second‐round 3DPCR program except that 1 ng pMD19‐Cytb DNA was used directly as a template. Similarly, Phusion‐based single 3DPCR was performed using 1 ng pMD19‐Cytb DNA and Cytb primers, but with 1 U high‐fidelity Phusion polymerase (NEB), according to the following program: 95 °C for 5 min, 35 cycles at 85–90 °C for 30 s, 58 °C for 30 s, 72 °C for 60 s and a final extension step of 72 °C for 10 min. For 3DPCR, only the products amplified with the least denaturation temperature were subjected to cloning and sequencing.

### Amplification, cloning and analysis of mtDNA mutations

Subclones of Ramos and Ramos‐A23 were generated by serial dilution in 96‐well plates and further expanded for at least 2 weeks before sequencing. Genomic DNA and mtDNA were prepared by DNeasy blood and tissue kit (Qiagen) from cultured cells and isolated mitochondria, respectively. For analysis of mutations, all of the amplifications were performed with Phusion polymerase (NEB). Phusion‐based 3DPCR amplification of Cytb has been described, except using mtDNA as a template. The PCR products of interest were purified with QIAquick Gel Extraction Kit (Qiagen), A‐tailed with Taq and cloned into TOPO (Thermo) or pMD19‐T vector (Takara) for bidirectional sequencing. The sequences were checked for quality and nucleotide changes using sequencher software (Gene Codes, Ann Arbor, MI, USA). Primers used for analyzing mtDNA in human lymphoma cells were: D‐loop (D‐loopF, 5′‐GAAAACAAAATACTCAAATGGGCC‐3′; D‐loopR, 5′‐GCTGTGCAGACATTCAATTGTTATT‐3′), 12s rRNA (12sF, 5′‐CCCATCCTACCCAGCACACA‐3′; 12sR, 5′‐CTGGTTCGTCCAAGTGCACT‐3′), 16s rRNA (16sF, 5′‐GCTAAACCTAGCCCCAAACC‐3′; 16sR, 5′‐GTTGGGTTCTGCTCCGAGGT‐3′); primers for analyzing IgH variable region in Ramos (VH4_Ldr, 5′‐ATGAAACACCTGTGGTTCTT‐3′; JH_Cons, 5′‐CTTACCTGAGGAGACGGTGACC‐3′) [24]; and primers for analyzing Cytb in chicken DT40 (chkCytbF, 5′‐ATGGCACCCAACATTCGAAA‐3′; chkCytbR, 5′‐CGATTGTGGGGAAGAGGATA‐3′).

### Data analysis and statistics

Analysis of mutation frequency and hotspot enrichment was performed as previously described [3, 25]. Statistical comparison of mutation frequencies was performed using Fisher’s exact test (one‐tailed), and the significance of AID hotspot and CpG motif enrichment was assessed using the binomial test. Welch’s two‐sample *t*‐test was used to compare independent means. All statistical analyses were performed using r software. *P* values <0.05 were considered significant.

## Results

### AID mutational activities in mec1, Ramos and Ramos‐A23 cells

**Fig. 1:**
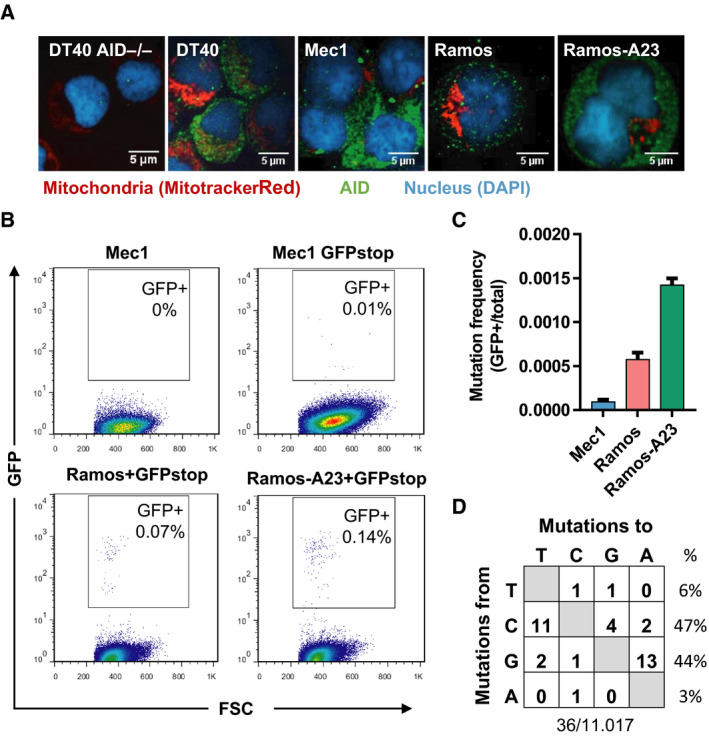
AID expression and mutational activity in Mec1, Ramos and Ramos‐A23 cells. (A) Confocal microscopy images of DT40 AID^−/−^, DT40, mec1, Ramos and Ramos‐A23 cells stained with anti‐AID antibody (green) and MitoTracker Red (red). Nucleus was counterstained with DAPI (blue). Scale bars: 5 µm. (B) Representative flow cytometry analysis of GFP reversion mutations. Mec1, Ramos and Ramos‐A23 cells were transduced with a GFPstop reporter construct and analyzed for GFP expression 21 days after transduction and puromycin selection. FSC, forward scatter. (C) AID mutational activity in Mec1, Ramos and Ramos‐A23 lines measured by reversion of GFPstop reporter. For each sample, at least 100 000 cells were analyzed, and mutation frequency was calculated by dividing the GFP^+^ count by the total number of counts. Data presented are mean and standard deviation of three biological replicates. (D) Mutation spectrum detected in the IgH variable region of Ramos cells. The total numbers of mutations and sequenced nucleotides are shown at the bottom.

To determine whether AID can target mtDNA, we used multiple B cell lines with constitutive AID expression, including human chronic lymphocytic leukemia line Mec1, human Burkitt lymphoma line Ramos and Ramos‐A23, a Ramos derivative that expresses high levels of AID [21, 26]. We also used chicken lymphoma line DT40 and its derivative carrying targeted deletion of AID (DT40 AID^−/−^) [22]. Immunofluorescence analysis confirmed the absence of AID in DT40 AID^−/−^ cells, moderate expression of AID in Ramos cells and abundant expression of AID in DT40, Mec1 and Ramos‐A23 cells (Fig. 1A). To confirm that AID can target nuclear DNA in these cells, we used a GFPstop reporter construct, which encodes an inactive GFP that can regain fluorescence upon AID‐mediated reversion of a premature stop codon, TAG [23]. We delivered the GFPstop reporter into Mec1, Ramos and Ramos‐A23 cells via retroviral infection, and measured the occurrence of GFP^+^ cells by flow cytometry (Fig. 1B,C). Although the protein level of AID is high, Mec1 cells exhibited the lowest AID mutational activity, which is consistent with the fact that the Mec1 line is derived from leukemic cells carrying unmutated Ig, possibly because of the high‐fidelity processing of AID‐induced C‐to‐U mutations in this line [26]. In comparison, Ramos and Ramos‐A23 cells exhibited much higher AID mutational activity than Mec1 cells, with the highest mutational activity observed in Ramos‐A23 cells, which correlates well with AID dosage and also is consistent with previous reports that Ramos cells constitutively mutate their Ig [21].

### Few mtDNA mutations in Mec1, Ramos and Ramos‐A23 cells

We next screened different mtDNA regions (D‐loop, 12s rRNA, 16s rRNA and Cytb gene) for mutation using mtDNA from purified mitochondria of Mec1, Ramos and Ramos‐A23 cells. For each region, an ~1‐kb segment was amplified, cloned and sequenced for point mutations, with around 40 kb nucleotides of sequence information collected for each gene. We identified dozens of preexisting single‐nucleotide polymorphisms (14 in Ramos and Ramos‐A23 and 21 in Mec1 cells; Table S1) that are clonal and differed from the mitochondrial reference sequence (rCRS) [27]. However, we identified only six nonclonal point mutations (C16223G and C33T in Ramos, C1352T and C15298T in Ramos‐A23, and C144T and A16524G in Mec1) in a total of 473 504 sequenced nucleotides (1.27 × 10^−5^ mutations/bp). In contrast, sequencing of the 0.5‐kb IgH variable fragment, the physiological target of AID in Ramos cells, revealed 36 point mutations in 11 017 sequenced nucleotides (3.3 × 10^−3^ mutations/bp; Fig. 1D). This surprisingly low level of mtDNA mutation in cell lines with different AID expression and mutational activity argued against a significant role for AID targeting of mtDNA.

### Development of high‐fidelity 3DPCR to detect rare mtDNA mutations

**Fig. 2:**
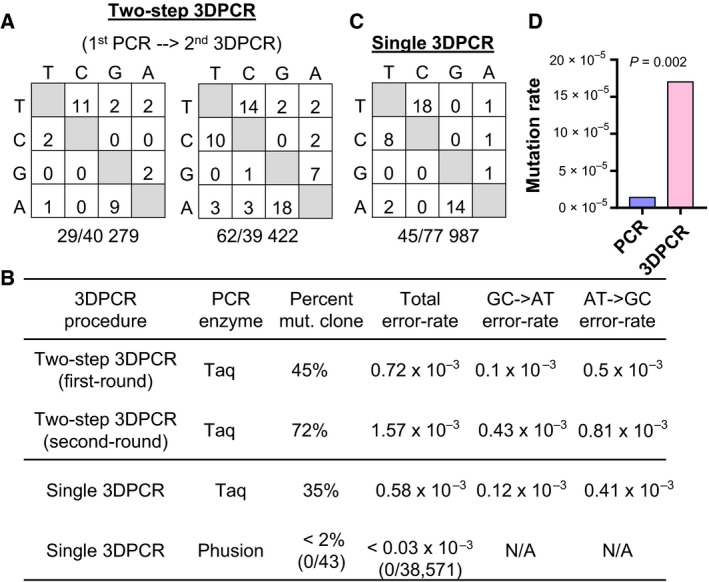
Development of high‐fidelity 3DPCR for detection of mtDNA mutations. (A) Error spectrum of Taq‐based two‐step 3DPCR using a plasmid template harboring an 897‐bp Cytb fragment. Errors generated during the first‐round standard PCR (left panel) and second‐round 3DPCR (right panel) were compared. In each panel, nucleotides in the left column are mutated to the nucleotides on the top row. The total numbers of mutations and sequenced nucleotides are shown below. (B) Summary of error rates for Taq‐based two‐step 3DPCR, Taq‐based single 3DPCR and high‐fidelity Phusion‐based single 3DPCR. The level of errors was comparable between single 3DPCR and standard PCR, with fidelity of polymerase being the major determinant. (C) Error spectrum of Taq‐based single 3DPCR using the same plasmid template as above. (D) Enrichment of Cytb mutations by Phusion‐based single 3DPCR. Cytb gene was amplified from mtDNA of Ramos‐A23 cells by either standard PCR or single 3DPCR, both with Phusion polymerase, and sequenced for mutations. A total of 144 kb sequencing data was obtained. Significance was assessed by Fisher’s exact test. The graph summarizes the data from three biological replicates.

The multicopy nature of the mitochondrial genome imposes a significant challenge for the detection and analysis of rare mtDNA mutations. Although 3DPCR can enrich mutations via biased amplification, Suspene *et al*. [28, 29] reported the cautious existence of high 3DPCR replication errors (4–20 errors kb^−1^). To enable the detection of rare mtDNA mutations, we first dissected the origin of 3DPCR errors in our context with a cloned 897‐bp fragment of mitochondrial Cytb gene using Suspene’s procedure, which consisted of first‐round standard PCR, followed by second‐round 3DPCR (two‐step 3DPCR) (Table S2). Sequencing of first‐round standard PCR revealed a total error rate of 0.72 kb^−1^ with ~45% of sequences (21 of 47) harboring 1–3 errors featuring A>G/T>C predominance, which is identical to the reported error rate and spectrum of Taq polymerase (Fig. 2A,C) [30]. The second‐round 3DPCR used the first‐round product as templates, which led to a doubling of error rate (1.57 kb^−1^) with 72% of sequences (33 of 46) harboring 1–4 errors (chi‐square test, *P* = 0.01; Fig. 2A,C). Notably, although the signature of A>G/T>C predominance was still evident, the G>A/C>T errors showed 4‐fold enrichment (0.1 versus 0.43 kb^−1^) during the second‐round 3DPCR, which is consistent with the continued accumulation of Taq polymerase errors and biased amplification of G>A/C>T errors generated during the first‐round PCR (Fig. 2A,C). We thus postulate that omitting first‐round PCR and using high‐fidelity polymerase may suppress 3DPCR errors. Indeed, Taq amplification of the cloned Cytb segment with single 3DPCR revealed an error rate and spectrum that was similar to standard PCR (Fig. 2B), whereas single 3DPCR with Phusion polymerase, which has more than 50‐fold higher fidelity than Taq, revealed zero errors in 38 571 sequenced nucleotides (Fig. 2C). We next tested amplification of Cytb with Phusion‐based single 3DPCR using mtDNA from Ramos‐A23 cells as a template. Further sequencing revealed 13 point mutations in 74 599 sequenced nucleotides, corresponding to a 12‐fold enrichment compared with the Phusion‐based standard PCR (Fig. 2D), suggesting that a Phusion‐based single 3DPCR approach can efficiently enrich mutations in mtDNA.

### Few AID targeting of mtDNA in AID‐high cells

**Fig. 3:**
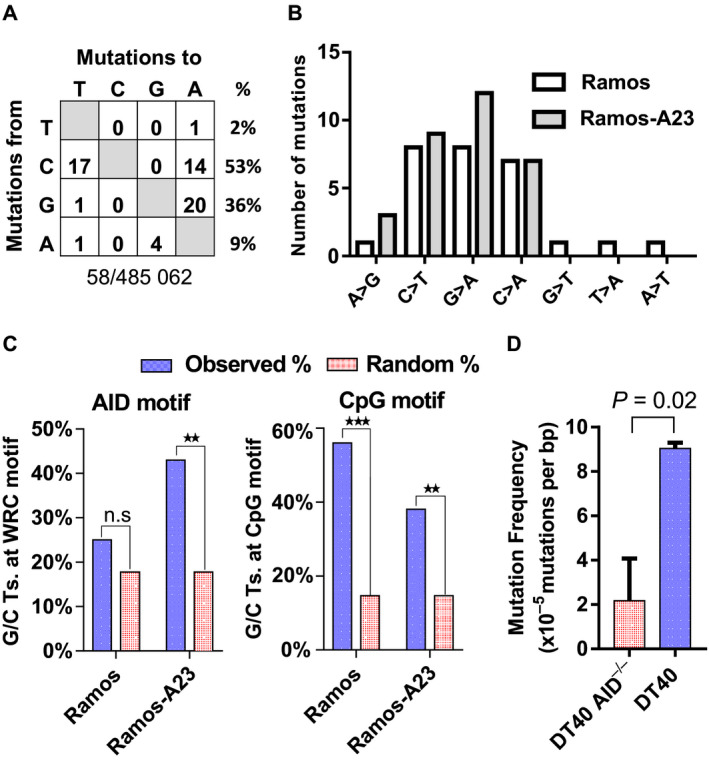
Weak targeting of mtDNA by AID. (A) Combined spectrum of Cytb mutations in Ramos and Ramos‐A23 cells recovered by Phusion‐based single 3DPCR. The total numbers of mutations and sequenced nucleotides are shown below. Percentage of mutations occurring at a specific base is calculated in the last column. (B) Comparable pattern of Cytb mutations recovered by Phusion‐based single 3DPCR in Ramos and Ramos‐A23 cells. The mutations are focused on G/C nucleotides, mostly C>T/G>A transitions and C>A/G>T transversions. (C) Distribution of Cytb mutations by AID hotspot motif (WRC/GYW) (left panel) and CpG dinucleotide motif (right panel). The mutation context of G/C transitions in Ramos (*n* = 3) and Ramos‐A23 cells (*n* = 3) was compared. Random % indicates the theoretical percentage of G/C transitions occurring at the AID motif or CpG motif upon random targeting, which is estimated based on the total number of G/C and the number of G/C within the AID or CpG motif. Significance was assessed by binomial test. **P < 0.01, ***P < 0.001; n.s., not significant. (D) Analysis of Cytb mutations recovered by Phusion‐based single 3DPCR using mtDNA from AID‐deficient or ‐proficient chicken DT40 lymphoma cells. The graph depicts the mean and standard deviation of mutation frequency from three biological replicates. Significance was calculated by Welch’s two‐sample *t*‐test.

We thus compared Cytb mutations in Ramos (AID‐low) and Ramos‐A23 (AID‐high) cells with a Phusion‐based single 3DPCR approach. Extensive sequencing of Ramos (228 819 sequenced nucleotides) and Ramos‐A23 cells (256 243 sequenced nucleotides) revealed a total of 58 point mutations (27 in Ramos and 31 in Ramos‐A23) (Fig. 3A and Table S3). Spectrum analysis revealed an abundance of G/C targeting mutations (G>A/C>T transition and C>A/G>T transversion) and paucity of A>G/T>C transitions, which distinguished them from the observed 3DPCR errors (Fig. 2A) and the reported Phusion amplification errors [30]. Furthermore, after correcting for base composition (guanine is 2.8‐fold less common than C in the Cytb fragment), the G>A transitions exhibited more than 3‐fold enrichment over C>T mutations (*P* < 0.001, binomial test). A similar trend of strand bias was evident for C>A/G>T transversions (14 C>A, 1 G>T). This mutational strand bias is also absent in 3DPCR errors but consistent with the heavy‐strand biased mutation signature in the mitochondrial genome [31, 32], confirming that these mutations are not PCR artifact but genuine mtDNA mutations.

Although Ramos‐A23 cells exhibited nearly 3‐fold higher AID mutational activity than Ramos cells (Fig. 1C), analysis of 3DPCR‐recovered Cytb mutations revealed comparable frequency (11.9 ± 3.5 × 10^−5^/bp and 13.4 ± 3.4 × 10^−5^/bp, respectively) and similar spectrums in both Ramos and Ramos‐A23 cells (Fig. 3B). AID has been well recognized for preferentially targeting C at WRC/GYW hotspot motifs (where W = A/T, R = A/G and Y = C/T) [33]. A closer analysis of mutation context revealed that only 25% of G/C transitions (4 of 16) in Ramos coincided with AID’s hotspot, which is expected by random targeting (binomial test, *P* = 0.5). However, 43% of G/C transitions (9 of 21) in AID‐high Ramos‐A23 cells was within the AID hotspot, which corresponded to a 2.4‐fold enrichment, supporting AID targeting of mtDNA in AID‐high Ramos‐A23 cells (binomial test, *P* = 0.007) (Fig. 3C). We also compared 3DPCR recovered Cytb mutations in AID‐high chicken lymphoma line DT40 (Fig. 1A) [22]. Sequencing of DT40 cells that were proficient or deficient of AID (DT40 versus DT40 AID^−/−^) revealed a 4.2‐fold higher mutation rate in AID‐proficient cells (Fig. 3D), further supporting AID targeting of mtDNA. However, the comparable mtDNA mutation rate between AID‐low Ramos and AID‐high Ramos‐A23 cells suggested that AID targeting of mtDNA is a rare event.

### Physical separation of AID from mtDNA

**Fig. 4:**
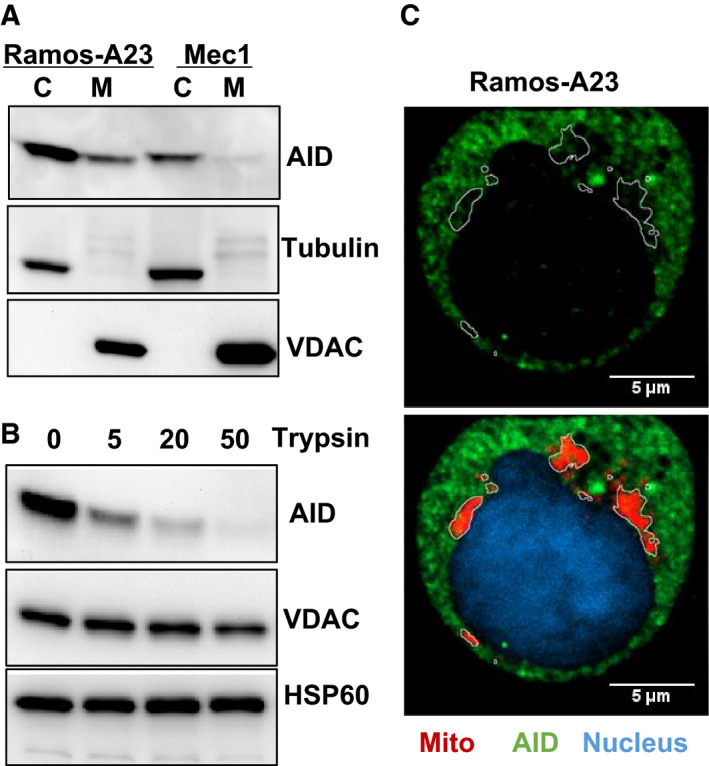
Physical separation of AID from mtDNA. (A) Western blot analysis of cytosol and mitochondrial AID proteins from Ramos‐A23 and Mec1 cells, showing that a fraction of AID is associated with mitochondria. The purity of proteins was assessed by cytosol‐specific Tubulin and mitochondrial outer membrane protein VDAC. (B) Mitochondrial‐associated AID is attached to the mitochondrial outer membrane. Purified Ramos‐A23 mitochondria were treated with different concentrations of trypsin (0–50 μg·mL^−1^) for 30 min on ice before immunoblot analysis. The integrated mitochondrial outer membrane protein VDAC and mitochondrial matrix protein HSP60 were used as control. All immunoblots were representative of three independent experiments. (C) Confocal microscopy analysis of AID–mitochondria colocalization. Ramos‐A23 cells were stained with AID antibody (green) and mitochondrial dye MitoTracker Red (red). Nucleus was counterstained with DAPI (blue). Mitochondrial contours were outlined at the bottom panel and overlaid onto the top panel. The marked mitochondrial areas were essentially free of AID staining. Scale bars: 5 µm. C, cytosol protein; M, mitochondrial protein.

To understand the mechanism underlying rare AID targeting of mtDNA, we tested whether AID can access mtDNA in the mitochondrial matrix. Western blot analysis of cytosol and mitochondrial fractions from both Ramos‐A23 and Mec1 revealed that AID is not exclusively confined within the cytosol proteins and a subset of AID is observed in the mitochondrial fraction (Fig. 4A). To further analyze AID’s submitochondrial localization, we treated isolated mitochondria with trypsin, which can remove the exposed proteins on the mitochondrial outer membrane without compromising outer membrane integrity [34]. As shown in Fig. 4B, trypsin digestion resulted in dose‐dependent loss of mitochondria‐associated AID, whereas integrated outer membrane protein VDAC and mitochondrial matrix protein HSP60 were not affected, suggesting that mitochondria‐associated AID is only externally attached to the mitochondrial outer membrane, but not located within the mitochondrial inner membrane space or matrix. In support of this, western blot analysis did not identify truncated AID variants, which would be expected for proteins with dual mitochondrial and cytosol localization, because the N‐terminal mitochondrial targeting peptide will be cleaved upon mitochondrial entrance. Consistently, immunofluorescence analysis revealed that the mitochondrial contour can clearly label areas that are essentially free of AID staining (Fig. 4C), supporting that AID cannot get access to mtDNA in the mitochondrial matrix.

### Recapitulation of endogenous mtDNA mutation signature by 3DPCR

Further analysis of Cytb mutations revealed that 56% of G/C transitions (9 of 16) in Ramos occurred within the CpG dinucleotide motif (3.8‐fold enrichment, *P* = 0.0001), whereas 38% of G/C transitions (8 of 21) in Ramos‐A23 were within the CpG motif (2.6‐fold enrichment, *P* = 0.007) (Fig. 3C), supporting the CpG motif as a mutational hotspot in mtDNA. This CpG mutation enrichment has also been observed in somatic mtDNA mutations across multiple human cancers [31, 35]. In addition, C>A/G>T transversions identified in both Ramos and Ramos‐A23 cells are hallmark patterns of oxidative DNA damage, consistent with mitochondria being the primary source of cellular reactive oxygen species [36]. Thus, Phusion‐based single 3DPCR also enabled us to capture at least three endogenous mtDNA mutation signatures: the mutational strand bias, the CpG mutational hotspot and the reactive oxygen species‐associated mutations.

## Discussion

The AID/APOBEC3 family proteins are single‐stranded DNA deaminases with key immunological functions. AID initiates somatic hypermutation and class switch recombination of Ig in the adaptive arm of immunity, whereas APOBEC3 members restrict virus infections in the innate arm of immunity [37]. In addition, off‐targeting of both AID and APOBEC3 members has been implicated as an important cause of genomic instability and cancer [4, 5, 38]. Because mitochondria play multiple critical roles and have their own mtDNA, a question of interest is whether mtDNA is targeted by these dangerous DNA mutators. Several APOBEC3 members, especially A3A and A3G, have been shown to be able to introduce a large number of point mutations into cytoplasmic mtDNA [39]. Our study here showed that AID can introduce very low levels of mtDNA mutations in AID‐high Ramos‐A23 and DT40 cells but cannot enter the mitochondrial matrix. As extensive literature has suggested the presence of mtDNA in the cytoplasm of cells [40, 41, 42], it is likely that cytoplasmic mtDNA is similarly targeted by AID, but at a much lower level. Thus, the mitochondrial genome is in general protected from AID/APOBEC3‐mediated mutagenesis by the physical segregation of AID/APOBEC3 mutators from accessing the mitochondrial matrix.

The 3DPCR technique was initially developed to detect viral genomes that are hypermutated by APOBEC3 proteins, because APOBEC‐edited DNA is richer in A+T and thus can be amplified at relatively lower denaturation temperature [28]. 3DPCR has also been extended to detect lightly mutated C>T/G>A transitions in cancer genomes [38, 43]. Recently, Suspene *et al*. [29] cast strong doubt on the application of 3DPCR beyond viral DNA editing by showing that 3DPCR can generate high levels of C>T/G>A errors at the rate of 4–20 kb^−1^. This high error rate was mainly estimated using low‐fidelity Taq polymerase and a two‐step 3DPCR procedure (first‐round standard PCR followed by second‐round 3DPCR). We here revisited the origin of errors in Taq‐based two‐step 3DPCR using a cloned 897‐bp Cytb fragment as a template. Our analysis showed that first‐round standard PCR generated an error rate of 0.72 kb^−1^ with mostly A>G/T>C and few C>T/G>A errors, which is identical to the error rate and spectrum of Taq polymerase [30]. Analysis of second‐round 3DPCR confirmed a higher error rate (1.57 kb^−1^) with 52% A>G/T>C and 27% G>A/C>T errors, which is consistent with the continued accumulation of Taq errors together with the biased amplification of G>A/C>T errors generated during the first‐round PCR. Thus, as shown here, Phusion‐based single 3DPCR amplification of Cytb is essentially error free and can successfully recover genuine rare mutations in mtDNA. The enrichment of A>G/T>C errors in our 3DPCR sequencing is not observed in Suspene’s analysis but may be explained by the difference in amplicon size. Compared with the 897‐bp Cybt template here, Suspene *et al*. [29] used much shorter templates (<300 bp), which may significantly magnify the amplification bias toward templates with G>A/C>T errors. Taken together, we propose that the level of 3DPCR errors is context dependent, with polymerase fidelity, amplification cycle and template size being three major determining factors. A limitation of Phusion‐based single 3DPCR is that Phusion cannot polymerize U‐containing template, but this can be overcome by a U‐compatible high‐fidelity polymerase, such as Phusion U or PfuTurbo Cx. In addition, Phusion can efficiently detect AID‐generated mutations in genomic DNA because AID‐generated U is rapidly processed by downstream low‐fidelity DNA repair pathways [44].

## Conflict of interest

The authors declare no conflict of interest.

## Author contributions

XG and MPS conceived the idea. XG performed the experiments, analyzed the data and wrote the paper. HW and KZ performed the experiments and analyzed the data. YC and JL cowrote the paper. All authors reviewed the manuscript.

## Supporting information


**Table S1**. List of clonal single‐nucleotide polymorphisms in the sequenced mtDNA regions of Ramos and Mec1 cells.
**Table S2**. List of 3DPCR errors generated by two‐step 3DPCR and single 3DPCR using cloned 897‐bp Ctyb template and Taq polymerase.
**Table S3**. List of 58 point mutations in Ramos and Ramos‐A23 subclones.

## Data Availability

The data that support the findings of this study are available from the corresponding author (XG) upon reasonable request.
