# Supplementary material for: Optimized high‐fidelity 3DPCR to assess potential mitochondrial targeting by activation‐induced cytidine deaminase
Source: FEBS Open Bio. 2020 Aug 13;10(9):1782–92. doi: 10.1002/2211-5463.12927 (PMC7459399; doi:10.1002/2211-5463.12927)
Supplement: Supplementary file 1 — Table S1. List of clonal single‐nucleotide polymorphisms in the sequenced mtDNA regions of Ramos and Mec1 cells. Table S2. List of 3DPCR errors generated by two‐step 3DPCR and single 3DPCR using cloned 897‐bp Ctyb template and Taq polymerase. Table S3. List of 58 point mutations in Ramos and Ramos‐A23 subclones. [file FEB4-10-1782-s001.pdf]

Supplementary information for

Optimized high-fidelity 3DPCR to assess potential mitochondrial targeting by activation-induced cytidine deaminase

Haiyan Wu1,2, Kaili Zhang1,3, Yue Chen3, Jinfeng Li2*, Matthew P. Strout4,□*, Xiwen Gu1*

1 Key Laboratory of Shaanxi Province for Craniofacial Precision Medicine Research, Research Center of Stomatology, Xi'an Jiaotong University College of Stomatology, Xi'an, Shaanxi, P. R. China

2 Department of Oral & Maxillofacial Surgery, Xi'an Jiaotong University College of Stomatology, Xi'an, Shaanxi, P. R. China

3 Department of Periodontology and Oral medicine, Xi'an Jiaotong University College of Stomatology, Xi'an, Shaanxi, P. R. China

4 Section of Hematology, Yale University School of Medicine, New Haven, CT, USA

□ Present address: Alexion Pharmaceuticals, New Haven, CT, USA

* Corresponding author

Jinfeng Li, jinfeng@mail.xjtu.edu.cn

Matthew P. Strout, matthew.p.strout@gmail.com

Xiwen Gu, xiwen.gu@xjtu.edu.cn

Supplementary Table 1. List of clonal SNPs in the sequenced mtDNA regions of Ramos and Mec1 cells.

Cell line	mtDNA position(rCRS)	SNPs	Locus
Ramos	73	A73G	D-Loop
Ramos	750	A750G	12S rRNA
Ramos	1438	A1438G	12S rRNA
Ramos	2092	C2092T	16S rRNA
Ramos	2706	A2706G	16S rRNA
Ramos	14766	C14766T	Cytb
Ramos	14783	T14783C	Cytb
Ramos	15043	G15043A	Cytb
Ramos	15301	G15301A	Cytb
Ramos	15326	A15326G	Cytb
Ramos	16223	C16223T	D-Loop
Ramos	16325	T16325C	D-Loop
Ramos	16362	T16362C	D-Loop
Ramos	16519	T16519C	D-Loop
Mec1	73	A73G	D-Loop
Mec1	143	G143A	D-Loop
Mec1	146	T146C	D-Loop
Mec1	750	A750G	12S rRNA
Mec1	813	A813G	12S rRNA
Mec1	1438	A1438G	12S rRNA
Mec1	2706	A2706G	16S rRNA
Mec1	14766	C14766T	Cytb
Mec1	14783	T14783C	Cytb
Mec1	15043	G15043A	Cytb
Mec1	15301	G15301A	Cytb
Mec1	15326	A15326G	Cytb
Mec1	16108	C16108T	D-Loop
Mec1	16129	G16129A	D-Loop
Mec1	16183	A16183C	D-Loop
Mec1	16189	T16189C	D-Loop
Mec1	16223	C16223T	D-Loop
Mec1	16249	T16249C	D-Loop
Mec1	16266	C16266T	D-Loop
Mec1	16311	T16311C	D-Loop
Mec1	16519	T16519C	D-Loop

Supplementary Table 2. List of 3DPCR errors generated by two-step 3DPCR and single 3DPCR using cloned 897bp Ctyb template and Taq polymerase.

3DPCR	Position[1]	Mutation	Contex[2]
Two-step / First-round	53	T>C	AATTA
Two-step / First-round	116	C>T	CTCCG
Two-step / First-round	221	T>C	CTTTT
Two-step / First-round	222	T>C	TTTTC
Two-step / First-round	226	T>C	CATCA
Two-step / First-round	286	A>G	CCAAT
Two-step / First-round	320	T>G	CCTAC
Two-step / First-round	323	A>G	ACACA
Two-step / First-round	338	T>A	CCTAT
Two-step / First-round	342	T>A	TATTA
Two-step / First-round	361	T>C	ACTCA
Two-step / First-round	374	A>G	AAACA
Two-step / First-round	399	A>G	GCAAC
Two-step / First-round	444	A>G	CAAAT
Two-step / First-round	468	A>G	GTAAT
Two-step / First-round	470	T>C	AATTA
Two-step / First-round	501	T>G	ATTGG
Two-step / First-round	501	T>C	ATTGG
Two-step / First-round	533	G>A	AGGCT
Two-step / First-round	612	A>T	GCAAC
Two-step / First-round	628	T>C	TATTC
Two-step / First-round	632	T>C	CTTGC
Two-step / First-round	639	A>G	GAAAC
Two-step / First-round	704	A>G	CTACA
Two-step / First-round	758	A>G	ATTAA
Two-step / First-round	764	T>C	ACTAT
Two-step / First-round	808	G>A	TAGCC
Two-step / First-round	831	C>T	CCCCA
Two-step / First-round	859	T>C	TATTC
Two-step / Second-round	39	C>T	ACCCC
Two-step / Second-round	47	G>A	ACGCA
Two-step / Second-round	68	A>G	AAAAT
Two-step / Second-round	91	G>A	TCGAC
Two-step / Second-round	110	A>G	CAACA
Two-step / Second-round	174	A>G	ACAGG
Two-step / Second-round	176	G>C	AGGAC
Two-step / Second-round	181	T>C	TATTC
Two-step / Second-round	187	G>A	TAGCC
Two-step / Second-round	226	A>C	CATCA
Two-step / Second-round	234	C>T	GCCCA
Two-step / Second-round	239	T>A	CATCA
Two-step / Second-round	249	C>T	GACGT

Two-step / Second-round	262	T>C	GCTGA
Two-step / Second-round	306	T>C	TTTAT
Two-step / Second-round	342	T>A	TATTA
Two-step / Second-round	347	G>A	CGGAT
Two-step / Second-round	365	A>G	AGAAA
Two-step / Second-round	367	A>G	AAACC
Two-step / Second-round	382	A>G	GCATT
Two-step / Second-round	396	T>C	CTTGC
Two-step / Second-round	410	C>T	AACAG
Two-step / Second-round	423	C>T	GGCTA
Two-step / Second-round	425	A>G	CTATG
Two-step / Second-round	428	T>G	TGTCC
Two-step / Second-round	433	C>T	TCCCG
Two-step / Second-round	435	G>A	CCGTG
Two-step / Second-round	454	T>C	TCTGA
Two-step / Second-round	478	T>C	ACTTA
Two-step / Second-round	479	T>C	CTTAC
Two-step / Second-round	485	C>A	ATCCG
Two-step / Second-round	487	G>A	CCGCC
Two-step / Second-round	495	A>T	CCATA
Two-step / Second-round	515	T>C	AGTTC
Two-step / Second-round	519	A>G	CAATG
Two-step / Second-round	528	A>G	TGAGG
Two-step / Second-round	535	T>C	GCTAC
Two-step / Second-round	543	A>G	GTAGA
Two-step / Second-round	553	A>G	CCACC
Two-step / Second-round	568	T>C	TCTTT
Two-step / Second-round	571	A>G	TTACC
Two-step / Second-round	581	T>C	CTTCA
Two-step / Second-round	608	T>C	CCTAG
Two-step / Second-round	615	A>G	ACACT
Two-step / Second-round	631	T>C	TCTTG
Two-step / Second-round	632	T>G	CTTGC
Two-step / Second-round	635	A>G	GCACG
Two-step / Second-round	645	A>G	GGATC
Two-step / Second-round	663	A>T	GGAAT
Two-step / Second-round	673	C>A	CCCAT
Two-step / Second-round	704	A>G	CTACA
Two-step / Second-round	723	C>T	CTCGG
Two-step / Second-round	738	C>T	TTCCT
Two-step / Second-round	750	A>T	TTAAT
Two-step / Second-round	751	A>G	TAATG
Two-step / Second-round	763	C>T	CACTA
Two-step / Second-round	787	G>A	GCGAC
Two-step / Second-round	796	A>C	ACAAT
Two-step / Second-round	800	A>G	TTATA
Two-step / Second-round	827	C>T	CCCTC

Two-step / Second-round	833	A>G	CCACA
Two-step / Second-round	766	T>C	TATTC
Single 3DPCR	69	A>G	AAATT
Single 3DPCR	80	A>G	CCACT
Single 3DPCR	115	T>C	TCTCC
Single 3DPCR	131	T>C	CTTCG
Single 3DPCR	157	A>G	TGATC
Single 3DPCR	165	A>G	CAAAT
Single 3DPCR	167	T>C	AATCA
Single 3DPCR	173	C>T	CACAG
Single 3DPCR	223	T>C	TTTCA
Single 3DPCR	235	C>T	CCCAC
Single 3DPCR	235	C>T	CCCAC
Single 3DPCR	265	A>G	GAATC
Single 3DPCR	320	T>C	CCTAC
Single 3DPCR	323	A>T	ACACA
Single 3DPCR	326	T>C	CATCG
Single 3DPCR	352	T>C	CATTT
Single 3DPCR	402	T>A	ACTAT
Single 3DPCR	442	C>A	GCCAA
Single 3DPCR	468	A>G	GTAAT
Single 3DPCR	555	C>T	ACCCT
Single 3DPCR	574	T>C	CCTTT
Single 3DPCR	600	T>C	ATTGC
Single 3DPCR	607	C>T	CCCTA
Single 3DPCR	712	A>G	TCAAA
Single 3DPCR	736	T>C	TCTTC
Single 3DPCR	740	T>C	CCTTC
Single 3DPCR	829	C>T	CTCCC
Single 3DPCR	838	A>G	TCAAG
Single 3DPCR	387	C>T	ATCCT
Single 3DPCR	415	T>C	CCTTC
Single 3DPCR	614	C>T	AACAC
Single 3DPCR	783	A>T	CTAGG
Single 3DPCR	122	G>A	ATGAT
Single 3DPCR	199	T>C	ACTCA
Single 3DPCR	213	A>G	TCAAC
Single 3DPCR	310	T>C	TCTGC
Single 3DPCR	338	T>C	CCTAT
Single 3DPCR	490	A>G	CCATC
Single 3DPCR	540	A>G	TCAGT
Single 3DPCR	568	T>C	TCTTT
Single 3DPCR	799	T>C	ATTAT
Single 3DPCR	801	T>C	TATAC
Single 3DPCR	833	A>G	CCACA
Single 3DPCR	849	A>G	TGATA
Single 3DPCR	868	A>G	ACACA

Note: 1) The mutated position relative to the cloned 897bp Cytb fragment.

2) The two nucleoties immedeiately 5' and 3' of the matated base were shown.

Supplementary Table 3. List of 58 point mutations in Ramos and Ramos-A23 subclones derived from single-cell-clonging.

Cell line	Subclones	Position[1]	Mutation	Context[2]	is_WRC	is_ROS	is_CpG
Ramos	1	14850	C>A	ctcac	N	Y	N
Ramos	1	14930	G>A	ccgcc	N	N	Y
Ramos	1	14831	G>A	ccgca	Y	N	Y
Ramos	1	14996	G>A	acgcc	N	N	Y
Ramos	1	14915	C>T	cacca	N	N	N
Ramos	1	14905	G>A	atgca	Y	N	N
Ramos	1	15045	G>A	acgag	N	N	Y
Ramos	1	15179	G>A	cagta	Y	N	N
Ramos	1	14860	C>T	ggcgc	N	N	Y
Ramos	1	14950	C>T	cacat	N	N	N
Ramos	1	14860	C>T	ggcgc	N	N	Y
Ramos	1	15285	C>A	tacct	Y	Y	N
Ramos	1	15505	A>G	ccaga	n.a	n.a	n.a
Ramos	1	14900	G>T	tagcc	N	Y	N
Ramos	2	15474	C>T	aacac	Y	N	N
Ramos	2	14945	G>A	tcgcc	N	N	Y
Ramos	2	14811	C>T	cccca	N	N	N
Ramos	2	15200	G>A	ccgcc	N	N	Y
Ramos	2	14832	C>A	cgcat	N	Y	N
Ramos	2	15004	C>T	ggcgc	N	N	Y
Ramos	2	14866	C>A	tgcct	Y	Y	N
Ramos	2	14989	C>A	tacct	Y	Y	N
Ramos	3	14962	C>A	gacgt	N	Y	Y
Ramos	3	14823	A>T	caaca	n.a	n.a	n.a
Ramos	3	14962	C>A	gacgt	N	Y	Y
Ramos	3	15453	T>A	ccttc	n.a	n.a	n.a
Ramos	3	14974	C>T	ggctg	N	N	N
Ramos-A23	1	14820	C>A	atcca	N	Y	N
Ramos-A23	1	14986	C>A	cgcta	N	Y	N
Ramos-A23	1	14974	C>T	ggctg	N	N	N
Ramos-A23	1	14906	C>T	tgcac	Y	N	N
Ramos-A23	1	14905	G>A	atgca	Y	N	N
Ramos-A23	1	14923	C>A	gcctc	N	Y	N
Ramos-A23	1	14944	C>A	atcgc	N	Y	Y
Ramos-A23	2	15093	G>A	cggca	Y	N	N
Ramos-A23	2	15464	A>G	taatg	n.a	n.a	n.a
Ramos-A23	2	14817	C>T	cccat	N	N	N
Ramos-A23	2	14930	G>A	ccgcc	N	N	Y
Ramos-A23	2	14920	C>A	gacgc	N	Y	Y
Ramos-A23	2	14996	G>A	acgcc	N	N	Y
Ramos-A23	3	14905	G>A	atgca	Y	N	N
Ramos-A23	3	15507	A>G	agaca	n.a	n.a	n.a
Ramos-A23	3	14996	G>A	acgcc	N	N	Y
Ramos-A23	3	14930	G>A	ccgcc	N	N	Y
Ramos-A23	3	15349	C>T	cacga	N	N	Y

Ramos-A23	3	15366	A>G	caacc	n.a	n.a	n.a
Ramos-A23	3	14995	C>T	cacgc	N	N	Y
Ramos-A23	3	15346	G>A	ttgca	Y	N	N
Ramos-A23	3	14950	C>A	cacat	N	Y	N
Ramos-A23	4	14923	C>T	gcctc	N	N	N
Ramos-A23	4	14905	G>A	atgca	Y	N	N
Ramos-A23	4	14905	G>A	atgca	Y	N	N
Ramos-A23	4	14905	G>A	atgca	Y	N	N
Ramos-A23	4	14860	C>T	ggcgc	N	N	Y
Ramos-A23	4	14888	G>A	cagga	N	N	N
Ramos-A23	4	14906	C>T	tgcac	Y	N	N
Ramos-A23	4	14995	C>T	cacgc	N	N	Y
Ramos-A23	4	14911	C>A	tactc	Y	Y	N

Note: 1) The mutated position relative to the mitochondrila genome.

2) The two nucleoties immedeiately 5' and 3' of the matated base were shown.

n.a, not applicable.
